# Abuse of Anabolic-Androgenic Steroids: Molecular and Organ Dysfunctions—A Critical Narrative Review for Forensic Interpretation

**DOI:** 10.3390/ijms27219730

**Published:** 2026-10-30

**Authors:** Donato Morena, Damiano Marchesini, Anna Maria Manta, Anna Claudia Caruso, Verdiana Santori, Pierluigi Crusco, Riccardo Sirello, Federica Umani Ronchi, Matteo Scopetti, Vittorio Fineschi

**Affiliations:** 1 Department of Anatomical, Histological, Forensic and Orthopedic Sciences, Sapienza University of Rome, 00161 Rome, Italy; donato.morena@uniroma1.it (D.M.); damiano.marchesini@uniroma1.it (D.M.); annaclaudia.caruso@uniroma1.it (A.C.C.); riccardo.sirello@uniroma1.it (R.S.); federica.umanironchi@uniroma1.it (F.U.R.); 2 Unit of Forensic Medicine, Azienda Ospedaliero-Universitaria Policlinico Umberto I, 00161 Rome, Italy; v.santori@policlinicoumberto1.it; 3 Department of Medical Surgical Sciences and Translational Medicine, Sapienza University of Rome, 00189 Rome, Italy; matteo.scopetti@uniroma1.it

**Keywords:** anabolic-androgenic steroid abuse, doping, forensic pathology, toxicology, spectroscopy, androgen receptor, cardiotoxicity, sudden cardiac death

## Abstract

Anabolic-androgenic steroids (AAS) comprise testosterone and synthetic androgenic compounds that differ substantially in pharmacokinetics, receptor activity, route of administration, hepatic metabolism, and clinical risk. From a forensic perspective, the central problem is not simply to catalogue adverse effects but to determine how verified exposure, chronology, co-use of other substances, clinical and electrocardiographic findings, pathological alterations, and toxicological results should be integrated when evaluating an individual death. This critical narrative review therefore distinguishes therapeutic testosterone replacement from non-medical and supraphysiological AAS exposure and examines the evidence connecting exposure to endocrine, cardiovascular, hepatic, renal, musculoskeletal, neuropsychiatric, and immunological findings. Human prospective and observational data support associations between androgen abuse and endocrine suppression, erythrocytosis, adverse lipid changes, increased blood pressure, and left-ventricular structural or functional alterations, with recovery of selected measured parameters reported in the studied cohorts. Electrocardiographic studies also report prolonged repolarization indices and impaired heart-rate recovery in AAS-using bodybuilders; however, these intermediate markers do not by themselves establish an individual risk of fatal ventricular arrhythmia. Forensic autopsy series describe myocardial hypertrophy, fibrosis, coronary disease, and other lesions in AAS-associated deaths, but these findings are not specific and require correlation with toxicology, clinical history, circumstances, and alternative causes. The detailed immunological literature is predominantly mechanistic, in vitro, or experimental and currently provides limited direct forensic evidence in human AAS users. Similarly, Raman spectroscopy is best regarded as a developing adjunct for forensic trace or analytical applications rather than a validated method for confirming AAS accumulation or cause of death in post-mortem tissue. We propose a structured forensic framework in which exposure is first verified, the temporal and pharmacological context is reconstructed, findings are assessed for specificity and plausibility, co-exposures and competing causes are examined, and the final opinion explicitly communicates the degree of certainty and the limitations of the evidence.

## 1. Introduction

Anabolic-androgenic steroids (AAS) are a chemically heterogeneous group that includes endogenous testosterone and synthetic derivatives developed to modify the balance between anabolic and androgenic effects [1].

Architecturally, synthetic AAS derivatives are categorized into three major chemical classes [2]:

- 17β-hydroxyl testosterone esters: Formulations such as testosterone enanthate, testosterone cypionate, and testosterone propionate, designed for sustained intramuscular release.
- 17α-alkylated derivatives: Compounds including oxymetholone, stanozolol, oxandrolone, and methandrostenolone (methandienone), engineered to withstand hepatic first-pass metabolism, which significantly increases oral bioavailability but escalates hepatotoxic potential.
- 19-nortestosterone modifications: Compounds such as nandrolone decanoate and trenbolone acetate, exhibiting heightened anabolic potency and distinct receptor binding dynamics. Drostanolone is a dihydrotestosterone derivative rather than a 19-nortestosterone derivative.

The historical literature describes therapeutic applications in selected endocrine, hematological, catabolic, and wasting conditions, whereas non-medical use has become established in competitive and recreational strength training [3]. Importantly, these exposure contexts are not interchangeable. The biological and clinical consequences of a medically supervised regimen intended to restore physiological testosterone concentrations cannot be extrapolated automatically to clandestine use of multiple AAS at supraphysiological exposure levels [4,5,6].

The distinction is clinically and forensically relevant. The Endocrine Society guideline defines hypogonadism using symptoms and signs of testosterone deficiency and unequivocally and consistently low testosterone concentrations, confirmed by repeat morning fasting measurements, and recommends diagnosis, indication, treatment targets, contraindication assessment, and monitoring of treatment response, serum testosterone and hematocrit [7]. In the TRAVERSE (Testosterone Replacement Therapy for Assessment of Long-term Vascular Events and Efficacy Response in Hypogonadal Men) randomized trial involving 5246 men aged 45–80 years with symptoms of hypogonadism and two fasting testosterone concentrations below 300 ng/dL, testosterone replacement with daily transdermal gel, titrated to maintain serum concentrations within a prespecified therapeutic range, was non-inferior to placebo for the primary major adverse cardiovascular event endpoint comprising cardiovascular death, nonfatal myocardial infarction, or nonfatal stroke in men with hypogonadism and pre-existing or high cardiovascular risk, although atrial fibrillation, acute kidney injury, and pulmonary embolism occurred more often in the testosterone group [8]. Non-inferiority for this composite endpoint should not be interpreted as the absence of all cardiovascular or other adverse effects, or as evidence of equivalent safety across testosterone formulations. These findings do not establish the safety of supraphysiological AAS misuse; rather, they demonstrate why therapeutic replacement and abuse must be treated as different exposures [7,8].

The relevant question is the following: how can verified AAS exposure be reconstructed and interpreted together with clinical, electrocardiographic, pathological, and toxicological findings in an individual case, while avoiding attribution based on non-specific lesions or on mechanistic evidence that has not been demonstrated in humans [4,6,9,10,11]?

## 2. Literature Identification and Evidence Appraisal

This manuscript is a critical narrative review rather than a systematic review or meta-analysis. The baseline evidence set was derived from the reference library of the submitted manuscript. During revision, targeted supplementary searches were performed for PubMed-indexed literature, including web-indexed searches of PubMed records, and on the websites of major medical societies and journal publishers to address the specific gaps identified during peer review, with targeted supplementary source verification updated on 9 October 2026. No lower publication-year limit was imposed. Search concepts included “anabolic androgenic steroids” or “androgen abuse” combined with cardiovascular disease, sudden cardiac death, electrocardiography, forensic autopsy, toxicology, hypogonadism, testosterone replacement, immune response, Raman spectroscopy, LC-HRMS (liquid chromatography–high-resolution mass spectrometry), and polypharmacy. Priority was given to human prospective studies, randomized trials or clinical guidelines where available, forensic case series and register studies, and analytical validation studies. Experimental, animal, and in vitro studies were retained primarily when they clarified mechanisms and were explicitly labelled as such.

Evidence was interpreted according to study type and forensic function. Human clinical studies were used to describe associations or intermediate phenotypes; forensic autopsy series were used to characterize patterns observed in fatal cases; case reports were used to illustrate possible clinical–pathological combinations rather than incidence; and experimental or in vitro studies were used to support biological plausibility. When evidence was conflicting, small, cross-sectional, self-reported, or substantially confounded by exercise or polypharmacy, the limitation is stated rather than resolved by inference. No new human or animal data were generated for this review.

Studies were considered eligible when they addressed human AAS exposure, AAS-related clinical or pathological findings, cardiovascular or other organ-system effects, forensic autopsy findings, analytical toxicology, or emerging analytical approaches relevant to forensic interpretation. Human prospective, observational, clinical, forensic, and analytical studies were prioritized. Case reports and case series were retained when they provided relevant forensic or pathological information, whereas animal and in vitro studies were included primarily when they contributed mechanistic evidence. Studies were excluded when they were unrelated to AAS exposure or did not provide information relevant to the clinical, pathological, toxicological, analytical, or forensic interpretation of AAS use. Study selection was performed by screening the identified literature for relevance to the predefined forensic question, followed by inclusion of sources considered most informative for the specific evidence domain. Because this was a critical narrative review rather than a systematic review, no formal PRISMA (Preferred Reporting Items for Systematic Reviews and Meta-Analyses) screening or quantitative study-selection procedure was undertaken. No independent duplicate screening, formal risk-of-bias tool or exhaustive retrieval is claimed. Supplementary queries included “anabolic androgenic steroids” with “cardiovascular disease”, “HAARLEM” (Health risks of anabolic androgenic steroid use by male amateur athletes), “forensic autopsy” or “toxicology”, and targeted title/DOI (digital object identifier) searches for the testosterone, immune-cell and Raman studies. Conflicting results were interpreted in relation to population, exposure and endpoint rather than pooled across incomparable designs.

## 3. Defining Exposure: Therapeutic, Non-Medical, and Supraphysiological Use

Forensic interpretation begins with exposure definition. Testosterone and synthetic AAS differ in formulation and metabolism, and their adverse-effect profiles are not identical. Oral 17α-alkylated compounds such as oxymetholone and stanozolol are particularly relevant to hepatotoxicity because structural modification increases oral bioavailability and alters hepatic metabolism. Long-acting injectable testosterone esters and other injectable AAS should not be treated as pharmacologically equivalent to these oral agents. Likewise, compounds such as nandrolone, trenbolone, and drostanolone differ in receptor activity, metabolism, and aromatization [2,6,8,10,12,13].

Oxandrolone provides a further example of why therapeutic and non-medical AAS exposure should be distinguished at the compound and clinical-context level. This synthetic 17α-alkylated anabolic steroid has been investigated as an adjunct in patients with severe burn injury, a setting characterized by profound hypermetabolism and catabolism. A systematic review and meta-analysis of clinical trials found that oxandrolone was associated with shorter hospital stay and improvements in weight regain, lean body mass, bone mineral density, and donor-site healing, without a demonstrated mortality benefit; the review did not show a significant increase in progressive or transient liver dysfunction, although liver-enzyme monitoring was recommended [14]. More recent evidence likewise supports potential benefits in metabolic recovery while emphasizing substantial heterogeneity across studies and the need for cautious interpretation [15]. These findings are specific to oxandrolone administered in defined burn-care populations and cannot be extrapolated to supraphysiological multi-agent AAS misuse. Conversely, therapeutic use should not be regarded as risk-free, and compound-specific adverse effects, dose, duration, patient characteristics, and monitoring remain relevant to forensic interpretation.

In clinical practice, therapeutic testosterone is prescribed for a defined indication and monitored with laboratory and clinical parameters. By contrast, AAS misuse commonly involves cycles, dose escalation, stacking of multiple AAS, and simultaneous use of other performance- or image-enhancing drugs. The HAARLEM cohort illustrates the heterogeneity of real-world exposure: participants used a wide range of cycles, doses, compounds, and co-exposures, and a substantial proportion of tested illicit products did not contain exactly what the label claimed. Such heterogeneity is a major reason why concentration, duration, and toxicity cannot be inferred from a single compound name alone [9,10,11].

For each investigated case, the exposure reconstruction should therefore document, whenever possible, (i) the exact compound or compounds; (ii) formulation and route; (iii) approximate dose and cycle duration; (iv) date of last administration; (v) therapeutic indications or absence thereof; (vi) co-used AAS and non-AAS substances; (vii) source and possible pharmaceutical quality of the product; and (viii) any clinical laboratory or monitoring data collected before death [7,8,16,17]. The exposure contexts and their interpretative implications are summarized in Table 1.

## 4. From Molecular Mechanism to Forensic Finding

**Figure 1:**
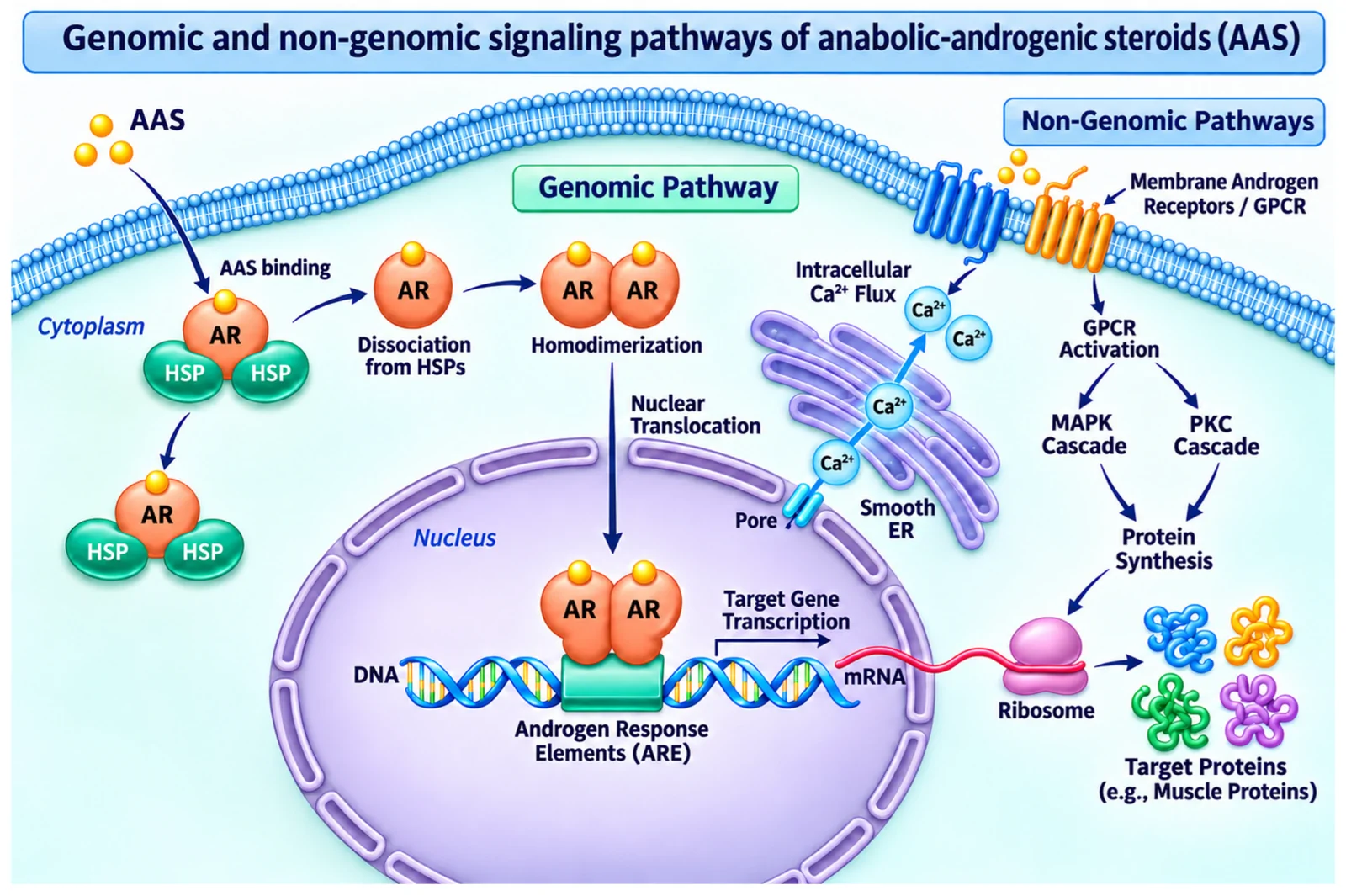
Genomic and non-genomic signaling pathways of anabolic-androgenic steroids (AAS). The genomic pathway involves androgen receptor (AR) activation, dissociation from heat shock proteins (HSPs), homodimerization, nuclear translocation, and interaction with androgen response elements (AREs), leading to changes in gene transcription. The non-genomic pathways illustrated involve intracellular calcium signaling and mitogen-activated protein kinase (MAPK) and protein kinase C (PKC) cascades. AI-generated schematic illustration. Abbreviations: GPCR (G protein-coupled receptor); ER (endoplasmic reticulum); DNA (deoxyribonucleic acid); mRNA (messenger ribonucleic acid); AI (artificial intelligence).

Most AAS exert their canonical effects through the androgen receptor (AR), a nuclear receptor that regulates transcription after ligand binding and nuclear translocation. Testosterone can also undergo tissue-specific conversion to dihydrotestosterone or oestradiol, further modifying the biological response. These mechanisms provide biological plausibility for changes in muscle, reproductive tissues, and metabolic or cardiovascular function, but a molecular mechanism is not equivalent to proof of causation in a specific death (Figure 1) [2,6]. The principal genomic and non-genomic pathways are illustrated schematically in Figure 1.

**Figure 2:**
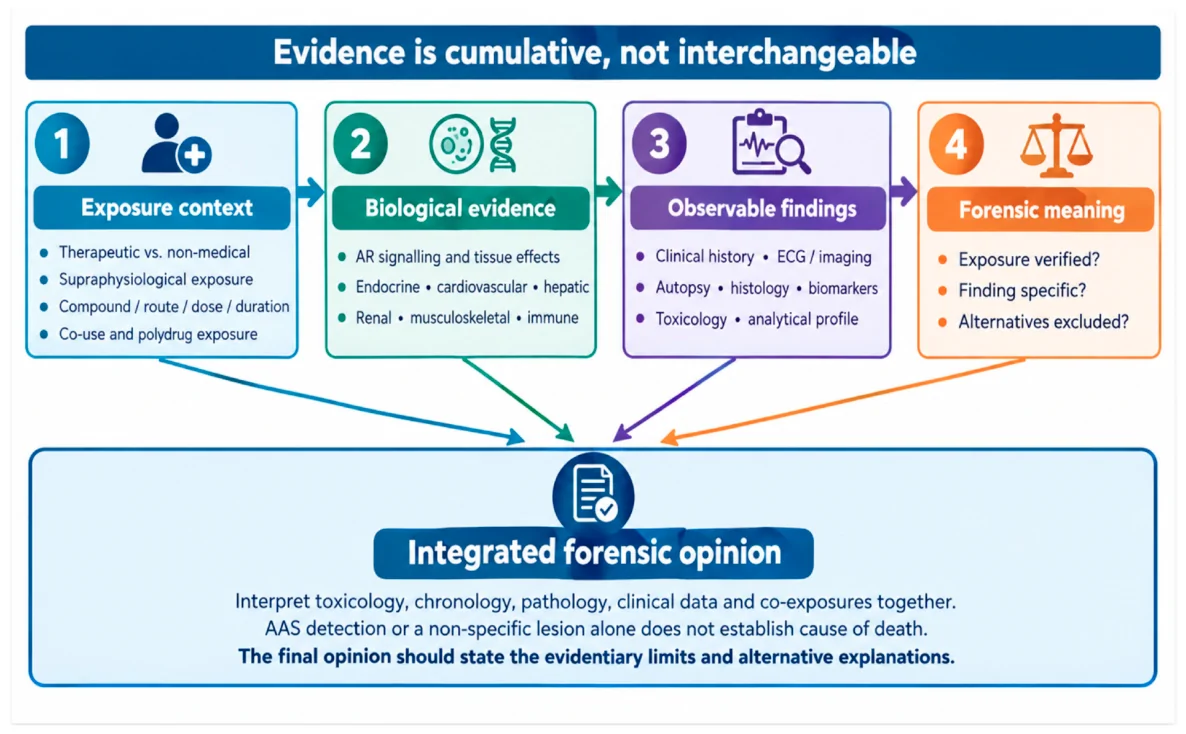
Original conceptual framework for forensic interpretation of suspected AAS-related findings. The sequence is exposure context → biological evidence → observable findings → forensic meaning. AI-generated schematic illustration.

The same distinction applies to rapid, non-genomic signaling. Calcium flux and kinase pathways have been proposed as contributors to acute cellular responses, yet their presence in experimental models does not establish that a post-mortem lesion is specific for AAS. For the purposes of forensic reasoning, molecular mechanisms are best used to connect a documented exposure with a plausible physiological effect, after which the relevant clinical, electrocardiographic, pathological, and toxicological observations must be independently established [2,6]. Figure 2 summarizes the proposed integration of exposure, biological evidence and observed findings within forensic interpretation.

## 5. Clinical, Pathological, and Forensic Findings by Organ System

Supraphysiological AAS exposure has been associated with alterations in several organ systems, with differing levels of supporting evidence. To standardize interpretation, each organ-system section follows the same sequence: exposure context, principal mechanisms, observed clinical or pathological findings, forensic relevance, and limitations. The detailed clinical-pathological manifestations are summarized below.

### 5.1. Reproductive and Endocrine Effects

Exogenous androgens suppress hypothalamic-pituitary-gonadal signaling through negative feedback. In men, prospective HAARLEM data from 100 men planning self-administered androgen cycles demonstrated marked suppression of endogenous gonadotropin and testicular function during androgen cycles, with recovery occurring on a longer timescale for spermatogenesis than for circulating testosterone. In women, androgen excess can produce virilizing effects, some of which may persist after exposure cessation [11,21].

In men, reduced gonadotropins, suppressed endogenous testosterone production, testicular volume reduction, and impaired spermatogenesis are compatible with recent or repeated androgen exposure but are not unique to illicit AAS use. Gynecomastia has been observed in androgen-abusing cohorts, but its persistence and severity vary. In women, menstrual disturbance, acne, hirsutism, voice deepening, and clitoral enlargement may accompany androgen excess [7,10,11,21].

Reproductive findings may support exposure reconstruction, especially when endocrine testing is contemporaneous with toxicological evidence. They are rarely sufficient to establish cause of death and should not be presented as specific markers of fatal AAS toxicity [11,17].

### 5.2. Hepatic Effects

Hepatotoxicity is particularly associated with orally active 17α-alkylated AAS. The available literature describes biochemical liver-test abnormalities and, more rarely, cholestatic injury, peliosis hepatis, adenoma, and carcinoma. However, severe clinical liver injury appears uncommon in prospective studies, and the incidence of rare tumors is not well defined [12]. A selected series of 44 men with bodybuilding-supplement-associated liver injury documented prolonged cholestasis; this characterizes an injury phenotype rather than its incidence among all users [22].

Mild to moderate elevations of aminotransferases may occur during AAS use, but interpretation is complicated in strength athletes because AST (aspartate aminotransferase) and ALT (alanine aminotransferase) are also expressed in skeletal muscle and may remain elevated after intense resistance exercise. In contrast, bilirubin and GGT (gamma-glutamyl transferase) may provide additional information when cholestatic injury is suspected. Reported cases of peliosis and hepatic tumors are important pathological observations, but they arise mainly from case reports and the older literature [6,12,22].

Liver pathology may corroborate a pharmacological exposure, particularly when a compatible 17α-alkylated agent is identified, but neither aminotransferase elevation nor non-specific hepatocellular injury establishes AAS causality. Product identity, co-medications, alcohol, viral hepatitis, metabolic disease, and exercise-related enzyme release should be evaluated [4,12,23].

### 5.3. Cardiovascular, Cerebrovascular, and Hematological Findings

This is the organ system with the strongest forensic signal in the current literature. Human prospective data from the HAARLEM cohort show that androgen cycles can increase blood pressure, worsen atherogenic lipid parameters, and increase hematocrit [24]. Echocardiographic follow-up also demonstrated increases in left-ventricular mass and transient deterioration in systolic and diastolic parameters during AAS cycles, with recovery after discontinuation in the studied cohort. Cross-sectional studies in long-term bodybuilders report altered autonomic function and ventricular repolarization indices [16,25,26,27]. The blood-pressure, lipid and hematocrit analysis involved 100 men [24]; the echocardiographic substudy included 31 men, of whom 25 were evaluated after recovery [18]. These HAARLEM reports derive from overlapping participants and should not be counted as independent replications.

Studies of AAS-using bodybuilders have reported lower heart-rate recovery after exercise and higher QTc, Tp-e, Tp-e/QT, or Tp-e/QTc values (QT, interval from the onset of ventricular depolarization to the end of ventricular repolarization; QTc, heart rate-corrected QT interval; Tp-e, T-wave peak-to-end interval). For example, a 2015 cross-sectional study compared 15 men using AAS for at least two years with 18 non-users; a 2024 study compared 21 AAS users with 23 non-users among 44 male bodybuilders [26,27]. Both reported differences in repolarization or heart-rate recovery measures. These findings are clinically interesting intermediate phenotypes, but the studies were small and did not establish that the measured ECG indices predicted a fatal arrhythmic event in an individual participant. The 2015 study explicitly noted the absence of observed arrhythmias and the need for longer follow-up [27]. When documented antemortem, such abnormalities may contribute functional evidence to the reconstruction of cardiovascular involvement, complementing structural findings at autopsy. Their interpretation requires consideration of co-used drugs, electrolyte disturbances and underlying cardiac disease, including inherited conditions when clinically indicated. They neither identify AAS as the specific cause of the abnormality nor establish the terminal cardiac rhythm. A Danish registry cohort comparing 1189 men sanctioned for AAS use with 59,450 population controls reported higher rates of several cardiovascular diagnoses over approximately 11 years. This supports a clinical association beyond surrogate markers, but residual confounding and incomplete exposure detail still limit individual attribution [28].

**Figure 3:**
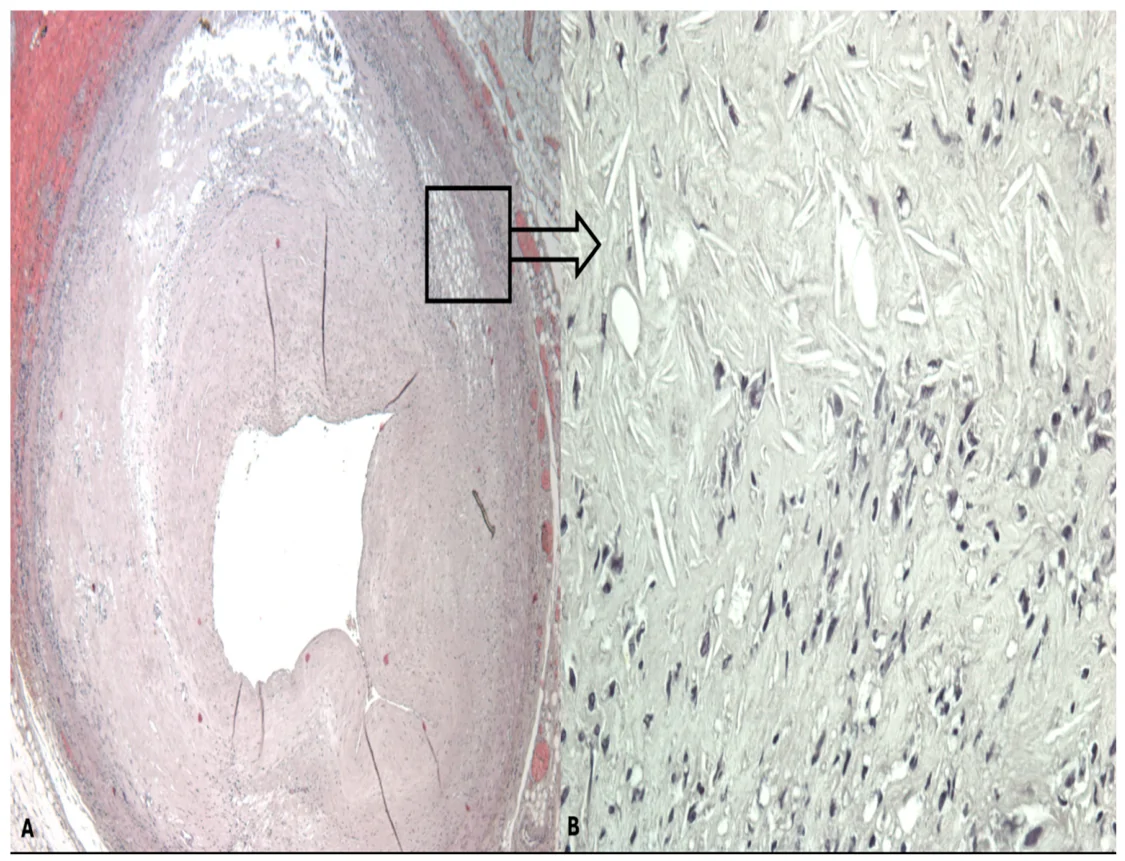
(**A**) Low-magnification cross-section of the left coronary artery demonstrating marked intimal thickening and severe luminal stenosis caused by an advanced atherosclerotic plaque in a 28-year-old bodybuilder (H&E, ×80). (**B**) High-power photomicrograph of the boxed region in (**A**) depicting the lipid-rich necrotic core, characterized by abundant needle-like cholesterol clefts (optically empty spaces left by dissolved cholesterol crystals during histological processing), accompanied by cell debris and inflammatory infiltrate (H&E, ×300). (Illustrative histological image from the authors’ archive.) Abbreviations: H&E (hematoxylin and eosin).

**Figure 4:**
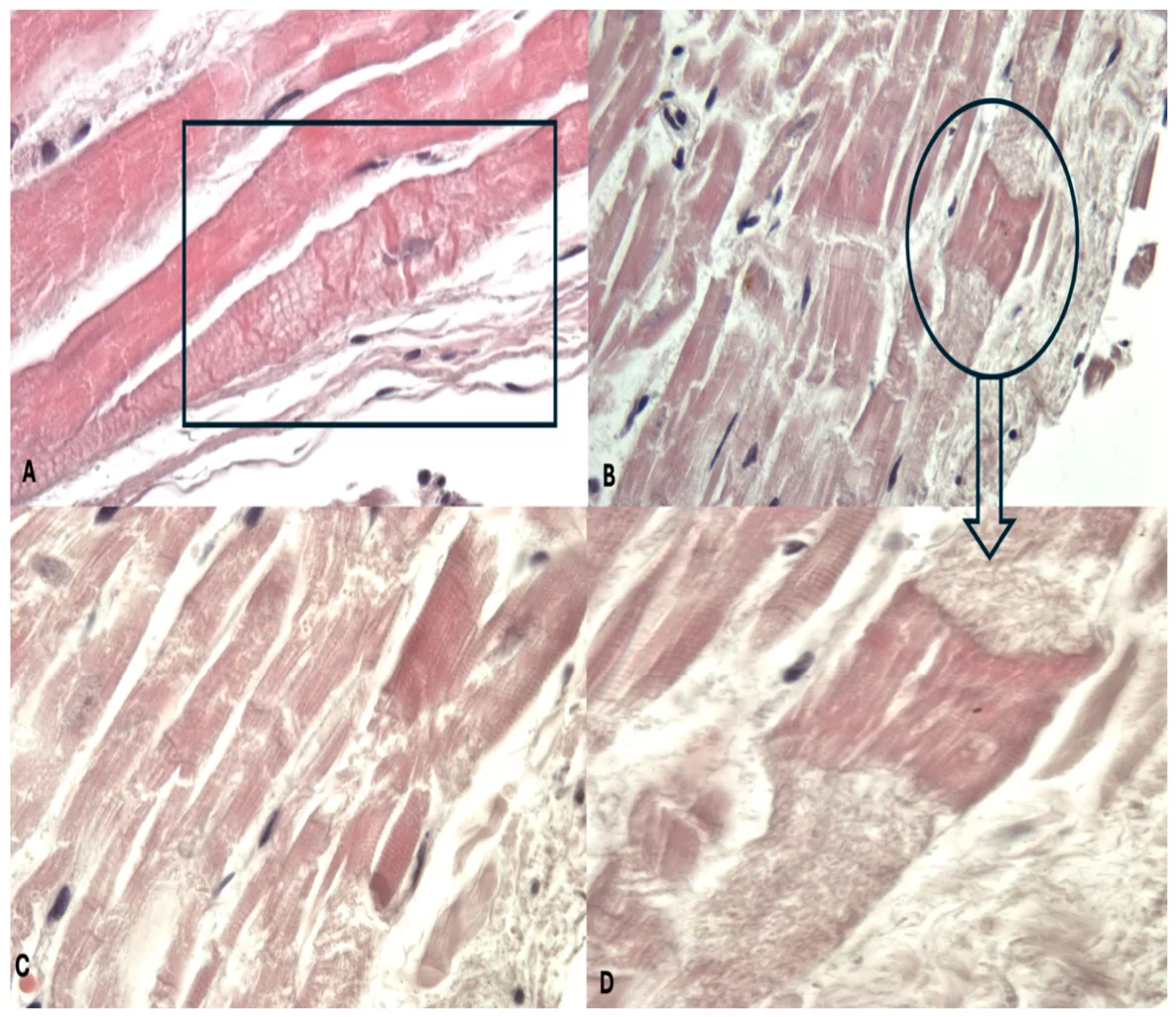
(**A**) Microscopic findings in a 28-year-old bodybuilder. Longitudinal view of a myocardial bundle: the rectangular inset showing pancellular lesion with fragmentation of hypercontracted myofibrils and band formation of hypercontracted or coagulated sarcomeres. Note the relative preservation of surrounding, less affected myocardial cells (H&E ×200). (**B**) Paradiscal lesion formed by about 15 hypercontracted sarcomeres without myofibrillar rhexis (H&E ×150). Black circle highlights the focus of early myocardial damage represented in high magnification in (**D**) with affected myocardiocytes show fragmented, granular sarcoplasm with dense eosinophilic (pink) bands and amorphous dense bodies, replacing the normal striated appearance. (H&E ×300). (**C**) Myocardium showing multifocal changes, including early fiber swelling and hypereosinophilia (H&E ×150). Illustrative histological image from the authors’ archive.

Forensic case series and reviews describe left-ventricular hypertrophy, myocardial fibrosis, myocyte disarray, contraction-band changes, coronary atherosclerosis or thrombosis, and cardiomyopathic remodelling in some deaths involving AAS. These lesions are biologically plausible in the context of chronic androgen exposure and are repeatedly reported in the forensic literature, but none are pathognomonic. Contraction bands can have multiple causes, fibrosis is a common final pathway of myocardial injury, and coronary atherosclerosis is influenced by age and conventional cardiovascular risk factors [29,30,31]. Figure 3 illustrates coronary atherosclerotic changes, while Figure 4 shows myocardial injury patterns.

In a suspected AAS-associated death, cardiovascular pathology becomes more informative when it is concordant with verified androgen exposure, a compatible chronology, antecedent hypertension or dyslipidaemia, ECG abnormalities recorded antemortem, and the exclusion of other major causes. A cardiac lesion should therefore be interpreted as part of an evidentiary pattern rather than as a stand-alone “AAS lesion” [15,29,30,31].

### 5.4. Musculoskeletal Findings

Rapid increases in muscle size and strength can occur with supraphysiological androgen exposure. The literature also contains reports of tendon injury in androgen users, including ultrastructural changes in ruptured tendons, but the evidentiary base is dominated by case reports and small observational studies [3,32,33]. A cross-sectional cohort study compared 88 AAS users with 54 non-users; its tendon-rupture findings rely on retrospective histories and remain subject to mechanical and behavioural confounding [32].

Tendon rupture may be relevant to the circumstances of death or injury but is not a specific toxicological finding. Nevertheless, comparative human evidence associates long-term AAS use with an increased occurrence of tendon rupture, particularly in the upper body, supporting its inclusion among clinically relevant musculoskeletal adverse outcomes of AAS abuse [32]. The proposed contribution of increased muscle force relative to tendon capacity illustrates how the anabolic effects of AAS may increase mechanical vulnerability even without a demonstrated, specific toxic lesion of the tendon [32].

It should be interpreted with training history, mechanical stress, prior tendon disease, age, and other exposures [32,33].

### 5.5. Renal Findings

Human evidence links long-term AAS abuse to clinically significant renal injury, although the independent contribution of direct nephrotoxicity remains less well established than the cardiovascular or endocrine effects. Herlitz et al. described ten bodybuilders with proteinuria and renal insufficiency, with biopsy-confirmed focal segmental glomerulosclerosis in nine. Seven patients who discontinued AAS and received renin–angiotensin system blockade showed stabilization or improvement of renal function and reduced proteinuria; one experienced recurrent proteinuria and renal insufficiency after resuming AAS abuse [34]. This temporal pattern supports a possible contribution of AAS exposure, although concurrent treatment and changes in body weight and lifestyle prevent isolation of its independent effect. Prospective androgen-abuse studies have reported modest and generally reversible changes in creatinine, while case reports and animal experiments have described proteinuria, glomerular injury, or rhabdomyolysis-associated acute kidney injury. Interpretation is complicated by high muscle mass, strenuous exercise, dehydration, creatine supplementation, and co-used substances [6,35,36].

Renal lesions or acute kidney injury may contribute to the reconstruction of a complicated death, but current evidence does not support treating them as specific indicators of AAS toxicity [6,35,36].

### 5.6. Neuropsychiatric and Infectious Considerations

Androgen abuse has been associated with mood and behavioural changes, including irritability, agitation, depressive symptoms after cessation, and other psychiatric presentations in susceptible individuals. The underlying evidence includes clinical studies, reviews, and case reports, and the effect of concurrent psychoactive substances is substantial. From a forensic perspective, psychiatric or behavioural observations are contextual rather than diagnostic of AAS exposure and should be reconstructed from medical and collateral records rather than inferred from the circumstances of death [5].

**Figure 5:**
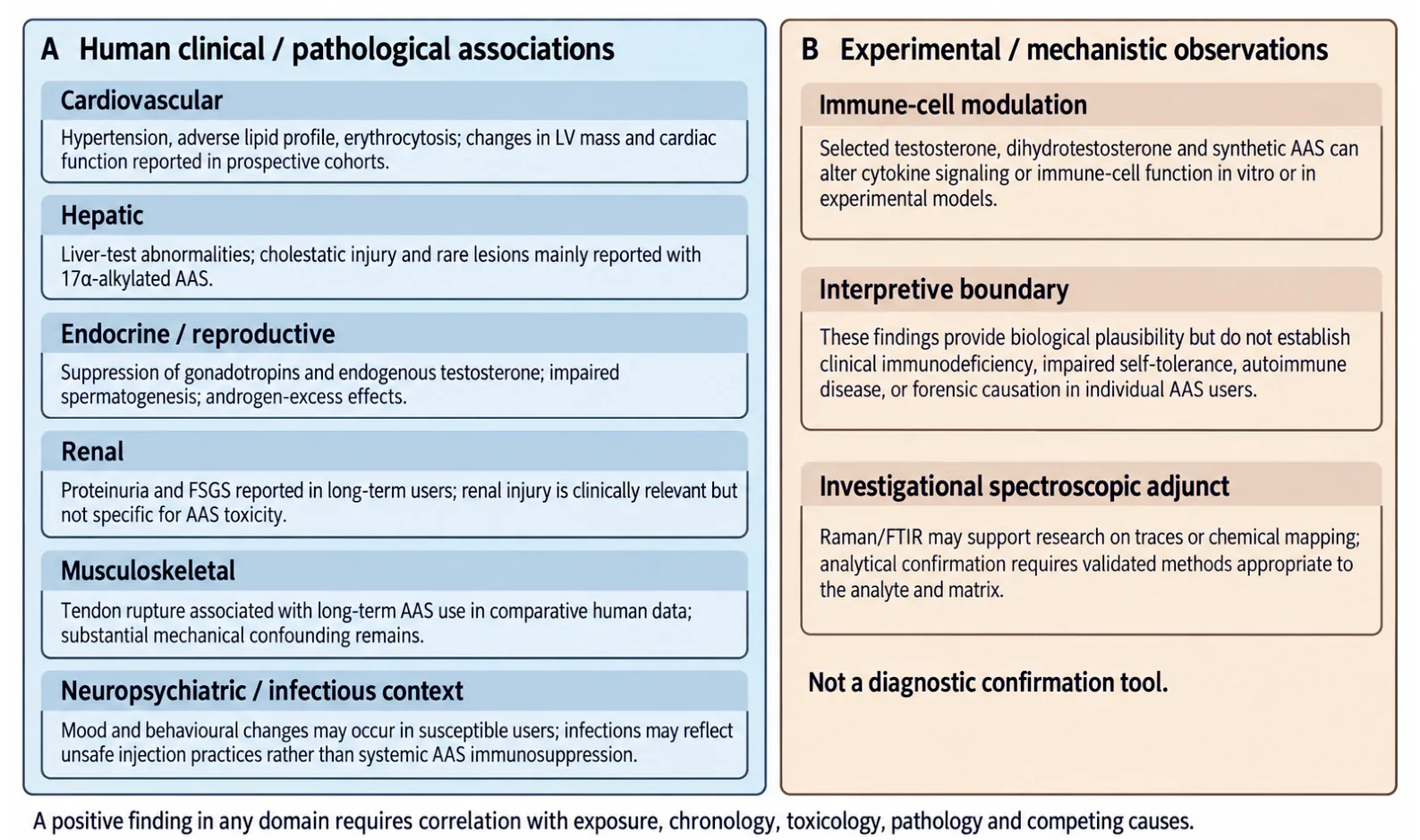
AAS-associated findings separated by evidentiary domain. Panel (**A**) summarizes clinical and pathological associations reported in human studies: cardiovascular [16,18,25,26,27,28], hepatic [12,22], endocrine/reproductive [11,21], renal [34,35,36], musculoskeletal [32,33], and neuropsychiatric/infectious findings [4,5,6]. Panel (**B**) presents selected experimental or mechanistic observations on immune-cell modulation [37,38,39,40,41,42] and an investigational spectroscopic adjunct [43,44,45]. The domains are not equivalent in forensic specificity, and none are independently diagnostic of AAS toxicity or cause of death. Abbreviations: LV (left ventricular); FSGS (focal segmental glomerulosclerosis); FTIR (Fourier-transform infrared spectroscopy).

Infectious complications should be conceptually separated from pharmacological immunomodulation. Injection with shared or non-sterile equipment and contaminated clandestine products can transmit blood-borne infections and cause local abscesses. Such events demonstrate unsafe administration practices or contaminated products; they do not establish systemic immunosuppression caused by AAS themselves [4,6]. Figure 5 provides a schematic overview of the organ-system findings discussed above, with the evidentiary qualifications indicated in its caption.

## 6. Immunological Evidence: Mechanistic Interest, Limited Forensic Specificity

Androgen receptors are expressed in immune cells, and experimental studies show that testosterone, dihydrotestosterone, and selected synthetic androgens can modulate cytokine signaling and immune-cell function. However, the populations, compounds, concentrations, endpoints, and biological systems differ widely across studies [37,38,39,40,41].

The evidence should therefore be separated into three categories. First, human observational studies have reported changes in selected immune parameters during androgen use, but these studies are limited in size and are difficult to interpret independently of exercise and other exposures [38]. Second, Marin et al. treated neutrophils from healthy male donors with testosterone at 10 nM, 0.1 µM and 10 µM for 24 h; the reduction in microbicidal activity reported at 10 µM is an isolated-cell finding and does not demonstrate clinical immunodeficiency in AAS users [42]. Third, animal and in vitro studies have identified compound- and model-specific effects on T-cell cytokine profiles and related immune functions. Hughes et al. reported that nandrolone decanoate induced IL-1β (interleukin-1 beta) and TNF-α (tumor necrosis factor-alpha) production in human peripheral blood lymphocytes in vitro and inhibited antibody production against sheep red blood cells in a murine model [37]. Dalal et al. reported a Th2 (T helper type 2) bias in an experimental autoimmune encephalomyelitis model following testosterone therapy, whereas Araneo et al. found that dihydrotestosterone reduced IL-4 (interleukin-4), IL-5 (interleukin-5) and IFN-γ (interferon-gamma) production by activated murine T cells [39,40]. These observations demonstrate that selected androgens can modulate immune-cell endpoints in defined experimental systems, but they do not establish a uniform effect across AAS compounds or a human clinical sequence from AAS use to loss of immune tolerance, infection susceptibility, or impaired tumor surveillance. Importantly, the cited evidence does not establish functional FoxP3+ (forkhead box P3-positive) Treg (regulatory T cell) depletion or generalized Th17 (T helper type 17) suppression as effects of AAS exposure. Nor should effects observed during concurrent aromatase-inhibitor administration be attributed to AAS themselves unless an independent AAS effect has been demonstrated [37,38,39,40,41,42].

**Figure 6:**
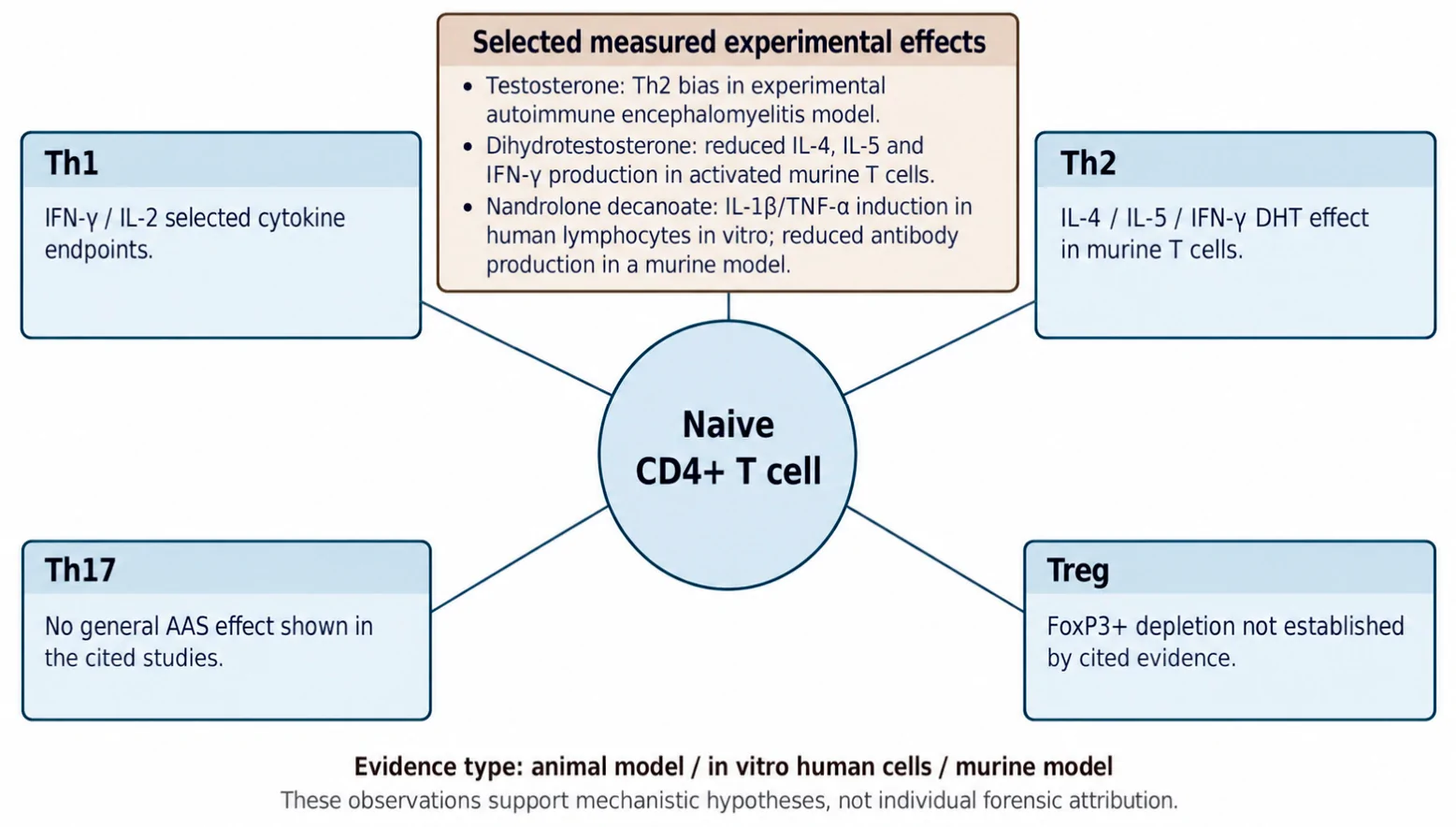
Experimental Androgen Effects on CD4+ T-cell Pathways. The scheme shows established CD4+ T-cell differentiation together with selected experimental androgen effects identified in the cited studies: testosterone-associated Th2 bias in an experimental autoimmune encephalomyelitis model [39]; reduced IL-4, IL-5 and IFN-γ production in activated murine T cells following dihydrotestosterone exposure [40]; and IL-1β/TNF-α induction in human lymphocytes in vitro and reduced antibody production in a murine model following nandrolone decanoate exposure [37]. Evidence type is explicitly indicated, and no downstream clinical or forensic diagnosis is inferred. Abbreviations: CD4 (cluster of differentiation 4); DHT (dihydrotestosterone); Th1 (T helper type 1); IL-2 (interleukin-2).

Accordingly, we do not use Th17 impairment, functional FoxP3+ Treg depletion, or reduced tumor surveillance as established forensic outcomes of AAS exposure. These mechanisms remain scientifically relevant as potential explanations for biological modulation and as targets for future translational research, but their current forensic utility in individual post-mortem cases is uncertain [37,38,39,40,41]. The proposed T-cell pathways are summarized in Figure 6 as mechanistic hypotheses rather than validated clinical or forensic endpoints. Figure 6 presents only these compound- and model-specific experimental observations and explicitly identifies the evidence type; effects of aromatase inhibitors are not presented as independent AAS effects.

## 7. Toxicology and Analytical Reconstruction of Exposure

AAS exposure should be established analytically whenever possible. Targeted LC-MS/MS (liquid chromatography–tandem mass spectrometry) and high-resolution mass spectrometry are well suited to identifying and quantifying parent compounds and metabolites in matrices such as whole blood and hair, while systematic toxicological analysis can identify co-used conventional drugs and other performance-enhancing substances. A validated LC-HRMS/MS (liquid chromatography–high-resolution tandem mass spectrometry) method has been reported for 12 steroids and related analytes in whole blood and 20 in hair, with low nanogram-per-millilitre detection limits in blood and picogram-per-milligram limits in hair and successful application to real cases [46]. More recent reviews confirm the continued importance of GC-MS (gas chromatography–mass spectrometry), LC-MS/MS, and LC-HRMS for targeted, suspect, and non-targeted steroid analysis [47].

The forensic question, however, is not simply whether an analyte is detected. Parent compound, metabolite profile, matrix, timing of sampling, route of administration, therapeutic use, and post-mortem processes influence interpretation. A positive result demonstrates exposure to the detected analyte or a compatible biotransformation pathway; it does not by itself demonstrate toxic concentration, pharmacological timing, or causal contribution to death. The possibility of counterfeit or mislabeled products further increases the value of broad analytical profiling [9,17,46].

Co-exposure is particularly important. A Finnish triple-register study of forensic autopsy cases found perimortem polysubstance use across AAS-positive and control groups, with a tendency toward greater central nervous system (CNS) drug polypharmacy and substantially more antemortem CNS prescription-drug purchases among AAS-positive cases. These findings support broad toxicological investigation but do not establish that any particular co-exposure caused death in an individual case [47,48]. Clenbuterol, thyroid hormones, selective estrogen receptor modulators, stimulants, recreational drugs, and other agents may contribute independent cardiovascular, neurological, metabolic, or thrombotic effects. Therefore, the toxicology strategy should be broad enough to identify the principal co-exposures rather than limiting the laboratory investigation to AAS alone [4,19,49,50]. Table 2 summarizes the roles and interpretative limitations of the main toxicological approaches.

## 8. Raman Spectroscopy and Other Spectroscopic Methods: An Adjunct, Not a Confirmatory Forensic Endpoint

Published forensic reviews support Raman spectroscopy as a useful, non-destructive approach for the characterization of a variety of forensic traces and biological evidence, while also noting limitations such as fluorescence, matrix interference, interpretation variability, and sensitivity constraints. A 2025 SERS (surface-enhanced Raman spectroscopy) study demonstrated rapid detection of selected AAS in food and pharmaceutical products, but this is an analytical study in non-human matrices and cannot be extrapolated to post-mortem human tissue [43,44,45].

In the present forensic framework, Raman or FTIR should therefore be described as proposed complementary techniques whose role depends on analyte, matrix, instrument configuration, spectral library quality, and independent validation. They may be useful for research into trace residues, counterfeit products, or chemical mapping, but they should not be presented as methods that confirm “AAS accumulation,” oxidative damage, or AAS-induced lethality in an individual death unless validated evidence demonstrating such performance becomes available [43,44].

## 9. Histopathology and Immunohistochemistry: What the Findings Can and Cannot Prove

**Figure 7:**
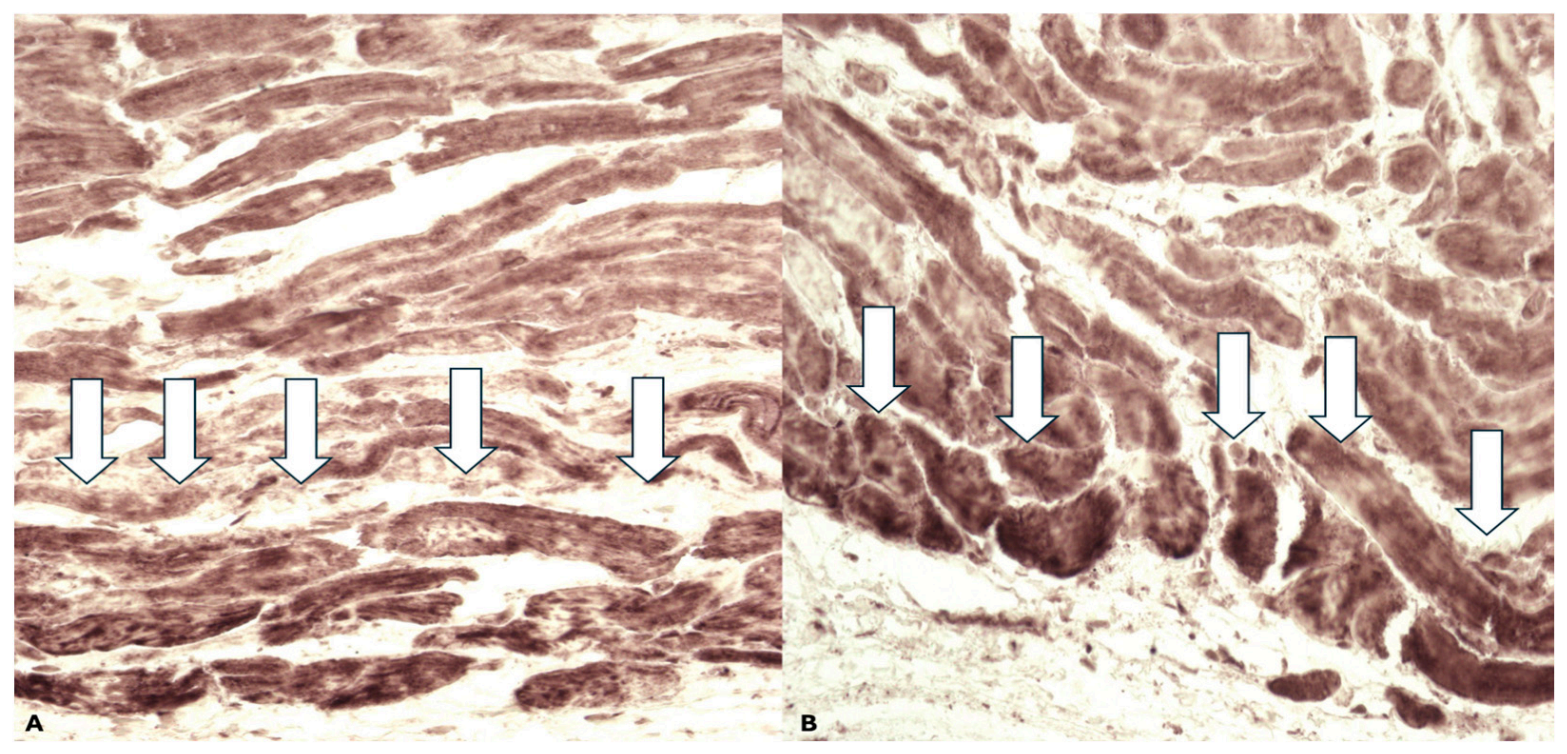
Myocardial Nuclear Factor kappaB (NF-κB) immunoreactivity. (**A**) Low-magnification (original magnification: 100×) view and (**B**) higher-magnification view showing myocardial immunopositivity. The image is presented as descriptive archival material; immunopositivity does not by itself establish pathway activation, AAS exposure, steroid toxicity, or cause of death (original magnification: 200×). Detailed case-level provenance and control information should be documented where available. Illustrative histological image from the authors’ archive.

**Figure 8:**
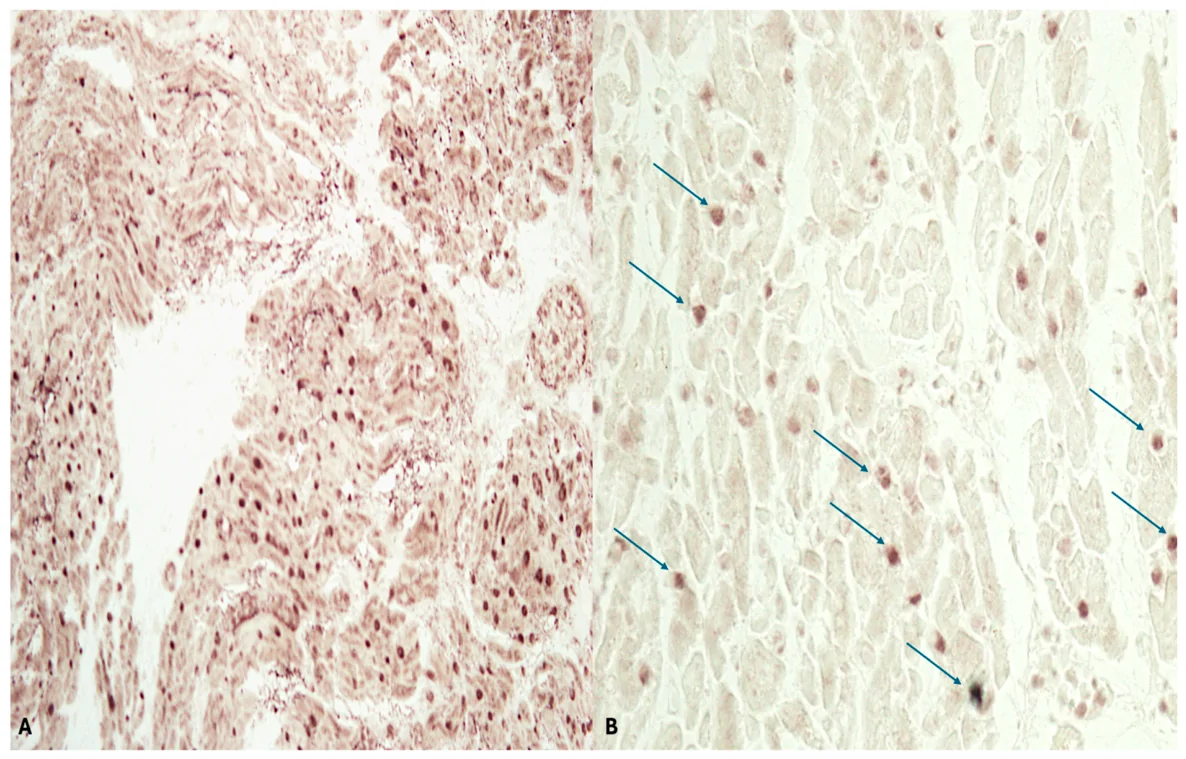
Myocardial BID and BCL2 immunoreactivity. (**A**) BID and (**B**) BCL2 staining in myocardial tissue. Two-panel composite of myocardial tissue sections stained for pro-apoptotic and anti-apoptotic proteins. (**A**) Immunohistochemical expression for BID, a pro-apoptotic member of the Bcl-2 family, shows distinct and diffuse brown immunopositivity within the myocardial cells. (Original magnification 100×). (**B**) Immunohistochemical staining for BCL2, an anti-apoptotic protein, also exhibits positive brown reaction product (indicated by thin arrows) (original magnification 200×). Illustrative histological image from the authors’ archive.

Histological findings are essential for excluding competing causes and documenting structural disease, but most changes described in AAS-associated deaths are non-specific. Myocardial hypertrophy, interstitial fibrosis, atherosclerosis, thrombosis, contraction bands, inflammatory staining, and apoptotic markers can be observed in multiple pathological settings. The evidentiary value increases when the lesion pattern is coherent with the clinical history, toxicological findings, and circumstances of death [17,29,30,51,52,53,54,55]. Figure 7 and Figure 8 illustrate myocardial immunoreactivity for NF-κB (nuclear factor kappa B) and BID/BCL2 (BID, BH3-interacting domain death agonist; BCL2, B-cell lymphoma 2; BH3, B-cell lymphoma 2 homology 3), respectively.

## 10. Practical Forensic Interpretation

Investigation should first reconstruct the compound, regimen, last use and co-exposures from toxicology, medical records and circumstances, including blood pressure, cardiovascular history and available antemortem ECG or imaging. The exposure chronology should distinguish a possible acute pharmacological effect from the contribution of disease associated with previous repeated use. Kintz et al. demonstrated that hair analysis could document multiple AAS not identified in blood collected from the same deceased individual [20]. Accordingly, failure to detect an exogenous AAS in post-mortem blood should not terminate the investigation of a possible chronic contribution when exposure history and cardiac findings warrant further assessment; the relevant question is whether prior exposure contributed to the disease underlying death, rather than whether an active compound remained detectable at death. Complete autopsy and representative histology should then establish disease and assess competing causes, including coronary disease, pulmonary thromboembolism, trauma and non-cardiac pathology. Histology and immunohistochemistry characterize lesions but do not independently identify AAS toxicity. This sequence is a practical synthesis of existing evidence, not a validated diagnostic score [9,46,56,57].

Peripheral blood and urine should be retained for targeted and broader toxicology; hair may complement exposure history but cannot establish an acute dose. Collect representative formalin-fixed tissue for histology and separate unfixed material for additional analytical, genetic or microbiological investigation when indicated. Record sampling site, timing, preservation and chain of custody, and agree collection requirements with the laboratory. These matrices have complementary purposes and require method-specific interpretation. Finally, integrate exposure and chronology with independently established pathology and alternative explanations, including polypharmacy. State whether AAS exposure is incidental, potentially contributory or of indeterminate relevance; neither detection alone nor exclusion of another cause establishes causation [20,29,30,46,50,56,58].

## 11. Limitations of the Evidence Base

The evidence base for AAS-related forensic attribution remains heterogeneous. Many human studies involve young male bodybuilders, limiting generalization to women, older adults, therapeutic users, and users of different compounds or combinations. Self-reported exposure is common, although prospective cohorts that chemically analyzed submitted products provide stronger exposure characterization. Cross-sectional studies can identify associations but cannot determine temporal direction or long-term clinical outcomes. Forensic case reports and autopsy series are valuable for identifying recurrent patterns but are inherently subject to selection and reporting bias. Experimental studies provide mechanistic plausibility while frequently using concentrations, routes, species, or exposure durations that do not match real-world human abuse [4,31,33,34].

Polypharmacy is a further major limitation [4]. AAS users may simultaneously use stimulants, β2-agonists, thyroid hormones, growth hormone, selective estrogen receptor modulators, recreational drugs, or prescription medications. Exercise itself changes blood pressure, cardiac adaptation, muscle enzymes, hydration, and other variables that complicate interpretation. Confounding should also be distinguished from mediation: hypertension and dyslipidemia may precede AAS exposure, but they may also develop as part of its cardiovascular effects. Statistical adjustment for these variables can attenuate estimates of effects mediated through them. In forensic interpretation, their presence should therefore prompt reconstruction of their chronology rather than automatic treatment as independent explanations that diminish the relevance of AAS exposure. Consequently, an AAS-related forensic opinion should rarely be based on a single finding or a single laboratory result [4,20,48,59].

## 12. Conclusions

The forensic significance of anabolic-androgenic steroid exposure lies in the integration of evidence rather than in the identification of a pathognomonic lesion [17]. The clearest human evidence supports an association between non-medical androgen use and endocrine suppression, adverse cardiovascular risk factors, erythrocytosis, and cardiac structural or functional changes, with recovery of selected measured parameters observed in the studied cohorts. Larger observational data also support associations with clinical cardiovascular events [28]. The forensic literature further documents myocardial remodeling and other cardiovascular lesions in AAS-associated deaths, but these findings are non-specific and should be interpreted in the context of confirmed exposure, chronology, co-exposures, clinical history, and alternative causes of death. Determining cause and manner of death in cases involving suspected AAS misuse requires a multi-disciplinary approach [53,58,60].

Immunohistochemistry may add mechanistic or phenotypic information in selected cases, but a positive marker such as NF-κB, BID, BCL2, or an altered structural protein cannot by itself establish steroid toxicity or cause of death [17,55].

A forensic conclusion is therefore not “AAS caused death” simply because an AAS is detected or because a cardiac lesion is present. The more appropriate approach is to demonstrate exposure, reconstruct the exposure history, document the observed phenotype and assess its compatibility with the exposure, identify or exclude competing explanations, and state transparently whether the available evidence supports a contributory role, remains indeterminate, or is insufficient for causal attribution [57,58,59,61].

## Figures and Tables

**Table 1:** AAS abuse: exposure contexts and forensic interpretation based on the cited literature.

Exposure Context	Typical Evidentiary Features	Interpretative Implication
Therapeutic testosterone replacement	Defined indication; physician supervision; dose adjusted toward physiological range; serial monitoring	Do not extrapolate findings from AAS abuse cohorts directly to this exposure [7,8].
Non-medical androgen use	Self-directed or non-prescribed use; variable compounds, doses and cycles; frequent co-use	Exposure reconstruction should rely on toxicology and contemporaneous history rather than assumptions about a standard “cycle” [4,6].
Supraphysiological/abusive exposure	High or repeated androgen exposure, often with stacking or ancillary drugs	The relevant comparator literature is AAS-abuse and androgen-cycle research, not routine replacement therapy [6,9,16].
Unknown/suspected exposure	Circumstantial clues, body habitus, injection sites, nonspecific pathology, or isolated analytical finding	Do not equate suspicion with confirmed exposure; prioritize targeted toxicology and chronology [17,18,19,20].

**Table 2:** Toxicology strategy and a methodological-reconstructive approach in cases related to the use of AAS: a synthesis of the cited literature.

Matrix/Approach	Primary Value	Important Limitation for Forensic Interpretation
Peripheral or femoral blood; targeted LC-MS/MS or LC-HRMS	Confirmation and, where validated, quantification of selected AAS and metabolites [46]	Concentration does not automatically equal toxicity; timing and post-mortem redistribution must be considered.
Urine	Evidence of metabolism and recent or prior exposure for compounds with informative metabolites [17,46]	Usually less suitable than blood for direct inference about circulating exposure at death.
Hair	Longer-term exposure history and detection of multiple compounds [20,46]	Segmental interpretation, external contamination, incorporation and cosmetic treatment may affect results.
Comprehensive toxicology	Identifies co-exposures, counterfeit products and alternative toxicological explanations [19,48]	A negative targeted panel does not exclude all androgen exposure, especially emerging or structurally modified compounds.
Immunoassay/screening	Rapid preliminary screening in some workflows [46]	Confirmation by a validated mass-spectrometric method is preferred when medico-legal attribution is required.

## Data Availability

No new data were created or analyzed in this study. Data sharing is not applicable to this article.
